# Predictive value of *K-ras* and *PIK3CA* in non-small cell lung cancer patients treated with EGFR-TKIs: a systemic review and meta-analysis

**DOI:** 10.7497/j.issn.2095-3941.2015.0021

**Published:** 2015-06

**Authors:** Jie-Ying Chen, Ya-Nan Cheng, Lei Han, Feng Wei, Wen-Wen Yu, Xin-Wei Zhang, Shui Cao, Jin-Pu Yu

**Affiliations:** ^1^Department of Immunology, ^2^Cancer Molecular Diagnostic Core Laboratory, ^3^Biotherapy Center, Tianjin Medical University Cancer Institute and Hospital, National Clinical Research Center for Cancer, Tianjin Key Laboratory of Cancer Immunology and Biotherapy, Tianjin 300060, China

**Keywords:** Non-small cell lung cancer (NSCLC), tyrosine kinase inhibitor (TKI), targeted therapy, *K-ras*, *PIK3CA*, meta-analysis

## Abstract

**Objective:**

A meta-analysis was performed to augment the insufficient data on the impact of mutative *EGFR* downstream phosphatidylinositol-3-kinase (*PI3K*) and mitogen-activated protein kinase (*MAPK*) pathways on the clinical efficiency of epidermal growth factor receptor tyrosine kinase inhibitor (EGFR-TKI) treatment of non-small cell lung cancer (NSCLC) patients.

**Methods:**

Network databases were explored in April, 2015. Papers that investigated the clinical outcomes of NSCLC patients treated with EGFR-TKIs according to the status of *K-ras* and/or *PIK3CA* gene mutation were included. A quantitative meta-analysis was conducted using standard statistical methods. Odds ratios (ORs) for objective response rate (ORR) and hazard ratios (HRs) for progression-free survival (PFS) and overall survival (OS) were calculated.

**Results:**

Mutation in *K-ras* significantly predicted poor ORR [OR =0.22; 95% confidence interval (CI), 0.13-0.35], shorter PFS (HR =1.56; 95% CI, 1.27-1.92), and shorter OS (HR =1.59; 95% CI, 1.33-1.91) in NSCLC patients treated with EGFR-TKIs. Mutant PIK3CA significantly predicted shorter OS (HR =1.83; 95% CI, 1.05-3.20), showed poor ORR (OR =0.70; 95% CI, 0.22-2.18), and shorter PFS (HR =1.79; 95% CI, 0.91-3.53) in NSCLC patients treated with EGFR-TKIs.

**Conclusion:**

*K-ras* mutation adversely affected the clinical response and survival of NSCLC patients treated with EGFR-TKIs. *PIK3CA* mutation showed similar trends. In addition to EGFR, adding *K-ras* and *PIK3CA* as routine gene biomarkers in clinical genetic analysis is valuable to optimize the effectiveness of EGFR-TKI regimens and identify optimal patients who will benefit from EGFR-TKI treatment.

## Introduction

Lung cancer remains the leading cause of cancer death in both genders according to the most recent statistics of the American Cancer Society^1^. Non-small cell lung cancer (NSCLC) accounts for more than 85% of lung cancers^2^. Most patients present with advanced NSCLC at the time of diagnosis, and chemotherapy becomes their palliative option. However, the poor improvement in the clinical response and survival outcomes of NSCLC patients who underwent chemotherapy over the last two decades highlights the need for more effective and less toxic treatments^3^.

Epidermal growth factor receptor tyrosine kinase inhibitor (EGFR-TKI) is a small-molecule drug that targets the active adenosine triphosphate binding site of EGFR kinase. Recent studies on patients bearing sensitive EGFR mutation have shown that EGFR-TKIs effectively increase clinical response rate and improve patients’ survival compared with standard chemotherapy, such as cisplatin plus gemcitabine or carboplatin plus paclitaxel, by inhibiting autophosphorylation and activation of downstream signaling pathways^4^^-^^7^. NSCLC patients harboring *EGFR* mutations benefit more from EGFR-TKI treatment than those without *EGFR* mutations. However, several studies demonstrated that gene mutations on the *EGFR* downstream signal pathways are also significant for the response of NSCLC patients to EGFR-TKIs.

*EGFR* activation elicits its effects via the *K-ras*/*BRAF*/mitogen-activated protein kinase (*MAPK*) and phosphatidylinositol-3-kinase (*PI3K*)/*AKT*/*mTOR* pathways, which promote tumor proliferation, invasion, migration, and neovascularization^8^. Mutation in the downstream genes of *EGFR* signaling pathways may result in receptor-independent pathway activation that renders the tumors unresponsive to EGFR inhibition. *K-ras* and *PI3K* are the key regulators on the two aforementioned pathways, respectively. *K-ras* encodes RAS, a guanosine triphosphate (GTP)-binding protein, which phosphorylates and activates MAPK by interacting with downstream *BRAF*, leading to a cascade of kinase reactions^9^. *K-ras* mutation attenuates the intrinsic GTPase activity of RAS protein, resulting in prolonged RAS activation^10^. The *PIK3CA* gene encodes the p110α catalytic subunit of PI3K protein, and its mutation leads to constitutive activation of protein kinase B signaling^11^. Both pathways play an important role in various cell physiological and pathological processes, such as proliferation, differentiation, apoptosis, and cell migration^12^^-^^14^. Although the corresponding frequencies of *K-ras* and *PIK3CA* mutations are approximately 5%-15% and 3%-5%^15^^,^^16^, many studies have reported that *K-ras* and *PIK3CA* mutations may have primarily induced resistance to EGFR-TKIs of NSCLC patients^17^^,^^18^.

A previous meta-analysis^19^ indicated a significant correlation between *K-ras* mutation and clinical response of NSCLC patients treated with EGFR-TKIs. However, the study merely focused on the objective response rate (ORR), and valuable information on the impact of *K-ras* mutation on the survival of NSCLC patients treated with EGFR-TKIs was not provided because of insufficient data. Similar studies on *PIK3CA* mutation are rarely reported. Thus, limited information on the clinical significance of gene mutations in the *EGFR* downstream signal pathways, especially for *K-ras* and *PIK3CA*, in NSCLC patients treated with EGFR-TKIs is available.

Therefore, we performed a meta-analysis of published studies to assess the impact of *K-ras* and *PIK3CA* mutation on the ORR, progression-free survival (PFS), and overall survival (OS) of NSCLC patients treated with EGFR-TKIs to clarify whether these mutations attenuate the clinical benefits of EGFR-TKI treatment in NSCLC patients.

## Materials and methods

### Search strategy

We developed a search strategy. An internet search of PubMed, EBSCO, OvidSP, and Wiley Online database was performed in April, 2015. Gefitinib and erlotinib, which are the first-generation EGFR-TKIs, had similar efficacies in NSCLC patients^20^^,^^21^. Thus, a combination of a disease domain (“lung cancer”), a treatment domain (“gefitinib”, “erlotinib”, or “EGFR TKI”), and a gene domain (“*kras*” or “*pik3ca*”) was used in all fields. The language was limited to English. All retrieved results were sent to EndNote software (EndNote X6, THOMSON REUTERS, US) to automatically and manually check for duplicate studies. After removing the duplicates, the titles and/or abstracts of the remaining results were screened to exclude irrelevant articles. Full texts of relevant articles were obtained and screened further for eligible studies. Bibliographies of relevant articles were hand-searched to determine additional eligible studies.

### Selection criteria

Two reviewers carefully and independently investigated all studies identified, and consensus was reached after discussion when disagreement in the inclusion or exclusion of studies was encountered. Inclusion criteria were as follows: (I) studies focused on NSCLC patients; (II) studies explored the relation between mutations of *K-ras* or *PIK3CA* and outcomes of NSCLC patients treated with EGFR-TKIs; and (III) studies assessed anti-tumor response using one or more of the following parameters: ORR, PFS, and OS. Distinguishing the predominant effect of the EGFR-TKI treatment was difficult when patients underwent combined therapy treatment. Therefore, exclusion criteria were as follows: (I) patients were not treated with single EGFR-TKIs; and (II) PFS and OS were not calculated from the initiation of EGFR-TKI treatment. When the same patient population was used in several publications, only the most recent, complete, or largest study was included in the meta-analysis.

### Data extraction

Data from all eligible studies were extracted independently by two researchers with disagreement settled by discussion. The following data from eligible studies were collected: publication details (such as the first author’s last name, publication year, and country in which the study was performed), trial information (such as inclusion criteria, number of patients assessed, therapy regimens, genes detected and detection methods, and type of end points used), patient characteristics (such as age, gender, stage, and histology), and outcome measures [such as hazard ratios (HRs) for PFS and OS and their 95% confidence intervals (CIs), log-rank test *P* values, and ORRs]. PFS and OS were defined as starting from the initial EGFR-TKI treatment. For PFS and OS, the HRs and their 95% CIs were estimated by methods proposed by Tierney *et al*.^22^ in the absence of published HRs or their 95% CIs. For ORRs, the reported number of objective response (complete response + partial response) and no response (progressive disease + stable disease) in each arm was collected. Quality was assessed independently by two investigators using the Newcastle-Ottawa scale (NOS) for non-randomized studies (available at http://www.ohri.ca/programs/clinical_epidemiology/oxford.asp) with consensus on all items through discussion.

### Statistical analysis

The relationship between gene mutation and ORR was presented by odds ratio (OR) with 95% CI. The impact of gene mutation on PFS and OS was measured by HR with 95% CI. The pooled ORs were computed for dichotomous variables by the Mantel-Haenszel method, and the pooled HRs and their 95% CIs were estimated by a general variance-based method. Heterogeneity across studies was tested by the *χ*^2^-based Q-test and *I*^2^ statistic. A *P* value greater than 0.10 for the Q-test and *I*^2^ statistic with values no more than 50% indicate the lack of heterogeneity among studies. Thus, the fixed-effect model was used for meta-analysis; otherwise, the random-effect model was used. Sensitivity analysis was conducted for meta-analyses by removing one study at a time to test the robustness of the overall results. Potential publication bias was estimated using Begg’s funnel plots and Egger’s linear regression test. All statistical tests were performed with STATA 12.0 (STATA Corporation, College Station, TX). All reported *P* values were two-sided. Differences were considered statistically significant at *P*<0.05.

## Results

### Literature search and study characteristics

**Figure 1:**
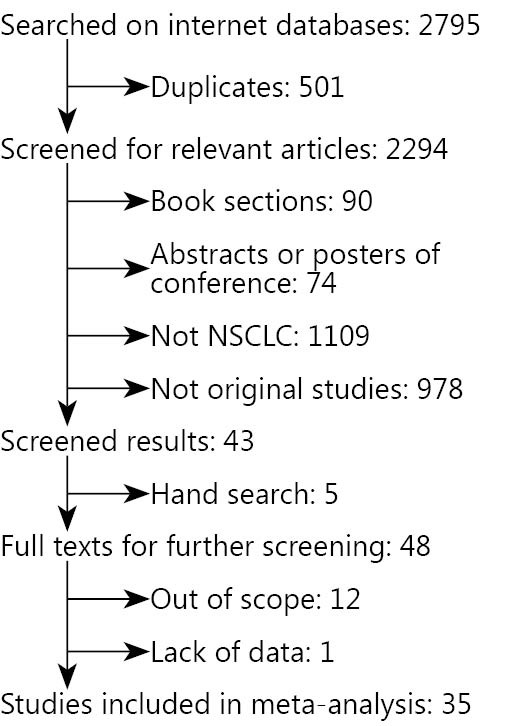
Flow diagram of selection process.

The initial search on PubMed, EBSCO, OvidSP, and Wiley Online database in April, 2015 retrieved 2,795 studies. A total of 2,294 articles remained after 501 duplicates were removed. After preliminary screening of titles and/or abstracts, 2,087 non-original or irrelevant studies, 90 book sections, and 74 abstracts or posters of conferences were excluded. Hand search on bibliographies of relevant articles retrieved five additional articles. Thus, full texts of 48 relevant studies were obtained for further investigation. Thirteen articles were further excluded because they were out of scope (12) and they lack relevant data (1). Finally, 19 articles^23^^-^^41^ published before 2010, 10 articles^17^^,^^18^^,^^42^^-^^49^ after 2010 and another 6 articles^50^^-^^55^ were included. The selection flow diagram is summarized in **Figure 1**.

The 35 studies were published from 2006 to 2014. These studies were conducted worldwide: nine from Italy^18^^,^^26^^,^^34^^-^^36^^,^^40^^,^^44^^,^^49^^,^^53^, five from multi-centers (more than two countries or regions)^33^^,^^39^^,^^42^^,^^51^^,^^52^, five from the United States^28^^,^^30^^,^^38^^,^^50^^,^^54^, two from Netherlands^29^^,^^48^, three from Japan^23^^,^^27^^,^^37^, two from Korea^17^^,^^25^, two from Germany^31^^,^^55^, and the rest were from Switzerland^24^, Greece^45^, France^43^, Czech Republic^47^, China^41^, Mexico^46^, and Taiwan^32^. The median age reported in 28 studies ranged from 58 to 75. A total of 3,958 patients were included with a mean sample size of 113 (ranged from 15 to 393). Most studies included patients with NSCLC, with only five studies focused on lung adenocarcinoma^17^^,^^35^^,^^36^^,^^49^^,^^50^, and one focused on lung squamous carcinoma^47^. Except in two studies^17^^,^^32^, all patients had inoperable stage IIIB or IV or recurrence. Previous treatments included chemotherapy, radiotherapy, surgery, or none. Current treatments of all included studies were monotherapy with EGFR-TKIs. In studies with treatment details, patients were treated with erlotinib or gefitinib according to international standard with one patient who received PF00299804, an irreversible TKI of EGFR, HER2, and HER4, in a study^17^. Clinical response was evaluated using RECIST criteria^56^ in 31 studies and WHO criteria^57^ in three studies, with one study not reported. Patients with complete or partial responses were classified as responders in all studies. ORR was the end point of 30 studies, PFS in seven studies, and OS in 11 studies. HR and corresponding 95% CI for PFS and OS were calculated from the primary data reported in the text of one study^17^, and estimated from the reported summary statistics with method recommended by Tierney in two studies^44^^,^^47^. The quality of all included studies was assessed with NOS. The quality scores of all studies were above 7, with mean score of 8.3.

### Biomarker analysis

A total of 33 studies provided the technological details for detecting gene mutations, and 16 studies performed mutation screening using direct sequencing (DS). The rest of the articles included pyrosequencing (1), denaturing capillary electrophoresis (DCE) (3), performance of amplification refractory mutation system (2), polymerase chain reaction (PCR)-restriction fragment length polymorphism (2), mutant-enrich sequencing (ME) (2), and denaturing high-performance liquid chromatography (2). A combination of the aforementioned methods was used in five studies. Mutation in *K-ras* exons 1, 2, and/or 3 was assessed in 34 studies, and *PIK3CA* exons 9 and/or 20 in 5 studies. Mutation of *EGFR* exons 18-21 was detected in all studies.

A total of 573 out of 3,377 evaluable patients were *K-ras*-mutation positive (17.0%), and 18 out of 473 patients were *PIK3CA*-mutation positive (3.8%). A total of 16 studies reported that *K-ras* mutation was mutually exclusive with *EGFR* mutation, and five other studies reported that 10 out of 178 patients positive for *K-ras* mutation were concomitant with *EGFR* mutation. Three studies reported that 6 out of 11 patients positive for *PIK3CA* mutation were concomitant with *EGFR* mutation. **Table 1** shows the main characteristics of studies included in the meta-analysis.

**Table 1:** Main characteristics of studies included in the meta-analysis

Reference	Location	No. of patients	Histology	Stage	Prior treatment	Current treatment	Gene	Mutation positive/total	End points	Response criteria	NOS score
Rotella 2014^49^	Italy	88	ADC	IIIB or VI	Surgery or chemotherapy or radiotherapy	E: 150 mg/d	*K-ras* (exon 2)	13/88	ORR	RECIST	8
Kim 2014^17^	Korea	55	ADC	I-IV	Chemotherapy	E, G, PANHER	*PIK3CA* (exon 9, 20)	3/55	ORR, PFS, OS	RECIST	7
Kerner 2013^48^	Netherlands	45	NSCLC	NR	NR	EGFR-TKI	*K-ras* (exon 2)	108/368	OS	RECIST	8
Fiala 2013^47^	Czech Republic	179	SLC	IIIB or IV	Chemotherapy	G: 250 mg/d or E: 150 mg/d	*K-ras* (codon 12,13) *PIK3CA* (exon 9)	14/174 6/170	PFS, OS	NR	9
Campos-Parra 2013^46^	Mexico	353	NSCLC	IIIB or IV	Chemotherapy	E or G	*K-ras* (codon 12, 13)	NR	PFS	RECIST	8
Murray 2012^45^	Greece	30	NSCLC	IIIB or IV or recurrent	Chemotherapy	G: 250 mg/d or E: 150 mg/d	*K-ras* (codon 12, 13)	3/30	OS	RECIST	8
Metro 2014^24^	Italy	67	NSCLC	IIIB or IV	Chemotherapy	E or G	*K-ras* (exon 2, 3)	18/67	ORR, PFS, OS	RECIST	9
Ludovini 2012^18^	Italy	166	NSCLC	III or IV	Chemotherapy	G: 250 mg/d or E: 150 mg/d	*K-ras* (exon 2, 3) *PIK3CA* (exon 9, 20)	11/162 6/145	ORR, OS	RECIST	9
Cadranel 2012^43^	France	307	NSCLC	Advanced	Chemotherapy	E: 150 mg/d	*K-ras* (exon 2)	42/307	PFS, OS	WHO	9
Hirsch 2011^42^	Multicenter	94	NSCLC	IIIB or IV	NR	E: 150 mg/d	*K-ras* (codon 12, 13)	20/94	ORR	RECIST	8
Zhu 2010^41^	China	17	NSCLC	IIIB or IV	NR	E: 150 mg/d	*K-ras*	3/17	ORR	RECIST	7
Tiseo 2010^40^	Italy	63	NSCLC	III or IV	Chemotherapy	G: 250 mg/d	*K-ras* (exon 2)	7/63	ORR	RECIST	8
Douillard 2010^39^	Multicenter	275	NSCLC	IIIB or IV	Chemotherapy	G: 250 mg/d	*K-ras* (codon 12, 13)	49/275	ORR	RECIST	9
Amann 2010^8^	USA	41	NSCLC	IIIB or IV or recurrent	Chemotherapy	E: 150 mg/d	*K-ras* (exon 2)	3/41	ORR, OS	RECIST	9
Varella-Garcia 2009^37^	Japan	30	NSCLC	Recurrent	Surgery	G: 250 mg/d	*K-ras* (codon 12, 13)	4/30	ORR	RECIST	9
Marchetti 2009^36^	Italy	83	ADC	IIIB or IV	Chemotherapy	G: 250 mg/d or E: 150 mg/d	*K-ras* (exon 2)	30/83	ORR, PFS, OS	WHO	8
Boldrini 2009^35^	Italy	411	ADC	IV	Surgery	G: 250 mg/d or E: 150 mg/d	*K-ras* (codon 12, 13)	2/19	ORR	RECIST	7
Zucali 2008^34^	Italy	49	NSCLC	III or IV	Chemotherapy or no	G: 250 mg/d	*K-ras* (exon 1, 2)	15/49	ORR	RECIST	8
Zhu 2008^33^	Multicenter	206	NSCLC	IIIB or IV	Chemotherapy	E: 150 mg/d	*K-ras* (exon 2)	30/206	ORR	RECIST	9
Wu 2008^32^	Taiwan	53	NSCLC	IB-IIIB	Surgery	E or G	*K-ras* (exon 1,2)	1/53	ORR	RECIST	8
Schneider 2008^31^	Germany	393	NSCLC	IIIB or IV	Chemotherapy or radiotherapy	E: 150 mg/d	*K-ras* (exon 2,3)	17/114	ORR, PFS, OS	RECIST	9
Miller 2008^30^	USA	82	NSCLC	IIIB or IV	Chemotherapy or no	E: 150 mg/d	*K-ras* (exon 2)	18/80	ORR	RECIST	9
Felip 2008^55^	Germany	39	NSCLC	Advanced	Chemotherapy or no	E: 150 mg/d	*K-ras* (exon 2,3)	7/39	ORR	RECIST	8
Zandwijk 2007^29^	Netherlands	15	NSCLC	NR	Chemotherapy	G	*K-ras* (codon 2)	3/15	ORR	RECIST	8
Massarelli 2007^54^	USA	70	NSCLC	IIIB or VI	Chemotherapy or no	G: 250 mg/d or E: 150 mg/d	*K-ras* (exon 1)	16/70	ORR	RECIST	9
Loprevite 2007^53^	Italy	21	NSCLC	Advanced	Chemotherapy	G: 250 mg/d	*K-ras* (exon 2)	1/21	ORR	RECIST	8
Jackman 2007^28^	USA	41	NSCLC	IIIB or IV	Surgery or Radiotherapy	E: 150 mg/d	*K-ras* (exon 2,3)	6/41	ORR	RECIST	9
Ichihara 2007^27^	Japan	99	NSCLC	Advanced or recurrent	Surgery or chemotherapy	G: 250 mg/d	*K-ras* (exon 2)	8/87	ORR	WHO	8
Hirsch 2007^52^	Multicenter	138	NSCLC	III or IV	Chemotherapy	G: 250 m g/d or E: 150 mg/d	*K-ras* (exon 2)	36/138	ORR	RECIST	9
Cappuzzo 2007^26^	Italy	37	NSCLC	IIIB or IV	Chemotherapy or no	G: 250 mg/d	*K-ras* (exon 2)	1/37	ORR	RECIST	7
Hirsch 2006^51^	Multicenter	152	NSCLC	III or IV	Chemotherapy	G: 250 mg/d	*K-ras* (exon 2)	12/152	ORR	RECIST	9
Han 2006^25^	Korea	69	NSCLC	IIIB or IV	NR	G:250 mg/d	*K-ras* (exon 2)	9/69	ORR	WHO	9
Giaccone 2006^24^	Switzerland	53	NSCLC	IIIB or IV	Surgery or Radiotherapy	E: 150 mg/d	*K-ras* (exon 1, 2) *PIK3CA* (exon 9, 20)	10/25; 1/25	ORR	RECIST	9
Pao 2005^50^	USA	59	ADC	NR	NR	G: 250 mg/d or E: 150 mg/d	*K-ras* (exon 2)	9/47	ORR	RECIST	7
Endoh 2006^23^	Japan	78	NSCLC	Recurrent	Surgery or chemotherapy	G: 250 mg/d	*K-ras* (codon 12, 13, 61); *PIK3CA* (open reading frame)	7/78; 2/78	ORR, OS	RECIST	8

ADC: adenocarcinoma; SLC: squamous lung cancer; NSCLC: non-small cell lung cancer; NR: not reported; E: erlotinib; G: gefitinib; PANHER: PF00299804, an irreversible TKI of EGFR, HER2, and HER4; ORR: objective response rate; PFS: progression-free survival; OS: overall survival; No.: number of patients assessed; NOS: Newcastle-Ottawa scale

### Predictive value of *K-ras* mutation

**Figure 2:**
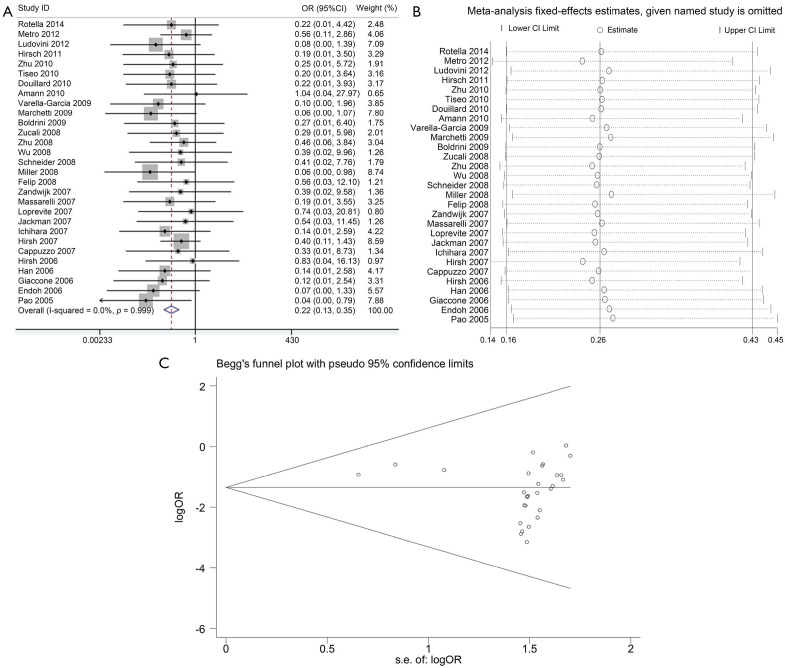
Meta-analysis of the predictive value of *K-ras* mutation for ORR. (A) Forest plots of OR and 95% CI; (B) Results of sensitivity analysis; and (C) Begg’s funnel plot analysis of publication bias. OR, odds ratio; ORR, objective response rate; s.e., standard error.

The impact of *K-ras* mutation on the ORR of NSCLC patients treated with EGFR-TKI therapy was evaluated based on 29 studies (**Table 2**). *K-ras* mutation was associated with reduced objective response in NSCLC patients with a pooled OR of 0.22 (95% CI, 0.13-0.35) (**Figure 2A**). Fixed-effect model was used because heterogeneity across the trials was not significant (*I*^2^=0%; *P*=0.999). The sensitivity analysis indicated that no individual study changed the pooled OR significantly (**Figure 2B**), suggesting that the result was reliable. Publication bias was significant in Begg’s test (*P*=0.049), but not in Egger’s test (*P*=0.090) (**Figure 2C**). Patients included in two studies^39^^,^^54^ apparently originated from the same center. Given that the independence of the two studies could not be confirmed, another analysis excluding the prior one of the aforementioned studies was conducted considering the possibility of duplicate patient population. The pooled OR was 0.22 (95% CI, 0.13-0.35) in a fixed effect model (*I*^2^=0%; *P*=0.998), with publication bias reduced significantly (*P* values in Egger’s and Begg’s tests were 0.101 and 0.072, respectively).

**Table 2:** Pooled results of meta-analysis of the predictive value of K-ras and PIK3CA mutation in patients with NSCLC

End points	No. of studies	Heterogeneity				Fixed model			Random model			Begg’s test			Egger’s test	
		*I*^2^ (%)		*P* value		OR/HR (95% CI)	*P* value		OR/HR (95% CI)	*P* value		Z	*P* value		*t* (bias)	*P* value
*K-ras*																
ORR	29	0.0	0.999			0.22 (0.13-0.35)	0.000		0.26 (0.16-0.43)	0.000		1.97	0.049		–1.76	0.090
PFS	6	0.0	0.748			1.56 (1.27-1.92)	0.000		1.56 (1.27-1.92)	0.000		1.13	0.260		2.86	0.046
OS	10	22.8	0.233			1.59 (1.33-1.91)	0.000		1.61 (1.31-2.02)	0.000		1.25	0.210		1.88	0.098
*PIK3CA*																
ORR	4	34.9	0.203			0.70 (0.22-2.18)	0.534		0.67 (0.12-3.62)	0.642		–	–		–	–
PFS	2	0.0	0.893			1.79 (0.91-3.53)	0.094		1.79 (0.91-3.53)	0.094		–	–		–	–
OS	4	26.9	0.255			1.83 (1.05-3.20)	0.034		1.82 (0.94-3.53)	0.075		–	–		–	–

ORR, objective response rate; PFS, progression-free survival; OS, overall survival; OR, odds ratio; HR, hazard ratio.

**Figure 3:**
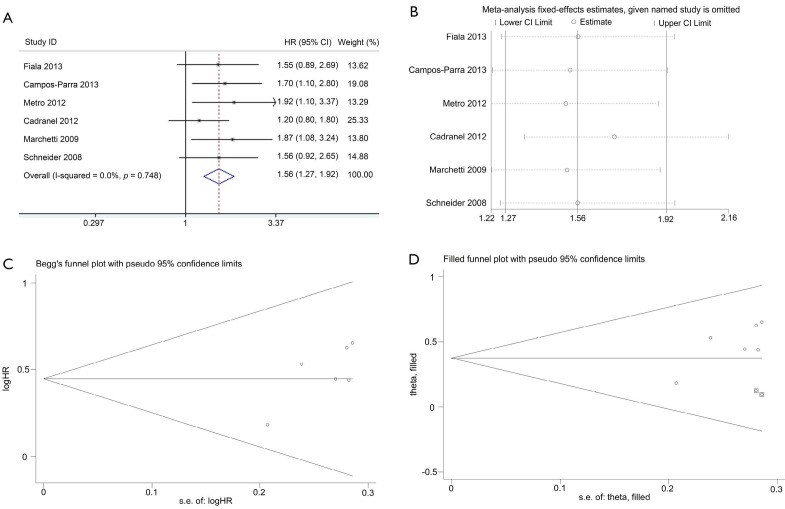
Meta-analysis of the predictive value of *K-ras* mutation for PFS. (A) Forest plots of HR and 95% CI; (B) Results of sensitivity analysis; (C) Begg’s funnel plot analysis of publication bias; and (D) Filled funnel plot using trim-and-fill method. ^○^, indicates observed studies; ◙, indicates missed studies. HR, hazard ratio; s.e., standard error.

Data for assessing the impact on PFS according to *K-ras* mutation status was available in six studies. *K-ras* mutant patients had shorter PFS compared with wild-type patients with pooled HR of 1.56 (95% CI, 1.27-1.92) (**Figure 3A**). Fixed-effect model was used when calculating pooled HR for PFS because heterogeneity across trials was not significant (*I*^2^=0%; *P*=0.748). Sensitivity analysis indicated that this result was robust (**Figure 3B**). Egger’s test revealed slight publication bias (*P*=0.046), contrary to Begg’s test (*P*=0.260) (**Figure 3C**). Thus, a non-parametric “trim-and-fill” method was utilized to adjust the publication bias (**Figure 3D**). After the trim-and-fill adjustment, two missing studies were added, and the estimated pooled HR was 1.46, with 95% CI ranging from 1.21 to 1.74.

**Figure 4:**
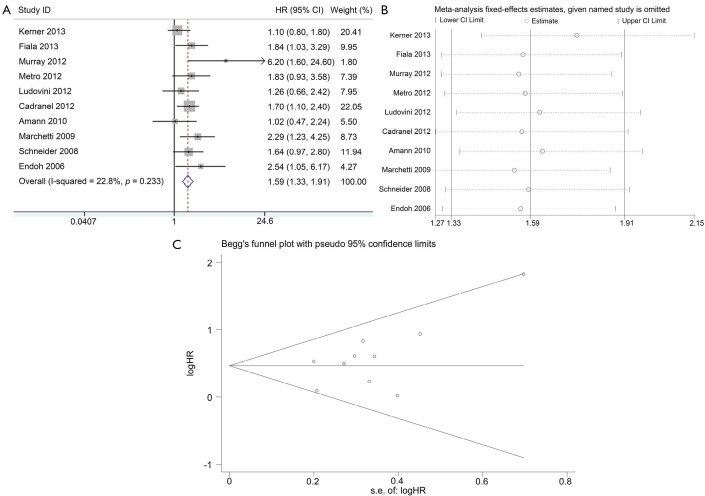
Meta-analysis of the predictive value of *K-ras* mutation for OS. (A) Forest plots of HR and 95% CI; (B) Results of sensitivity analysis; and (C) Begg’s funnel plot analysis of publication bias. HR, hazard ratio; s.e., standard error.

Ten studies were available for analyzing the impact on OS according to *K-ras* mutation. Results showed that NSCLC patients with *K-ras* mutation had shorter OS than wild-type patients with pooled HR of 1.59 (95% CI, 1.33-1.91) (**Figure 4A**). A fixed-effect model was used in calculating pooled HR for OS because heterogeneity across the trials was not significant (*I*^2^=22.8%, *P*=0.233). Sensitivity analysis indicated that the result was stable (**Figure 4B**). Publication bias was not significant in both Egger’s (*P*=0.098) and Begg’s tests (*P*=0.210) (**Figure 4C**).

To determine the slight heterogeneity across trials in the analysis of the impact of *K-ras* mutation on the OS of NSCLC patients treated with EGFR-TKIs, we conducted subgroup analysis based on whether *K-ras* mutation is concomitant with *EGFR* mutation, previous treatment, and mutation detection method (**Table 3**). Heterogeneity across trials decreased in most subgroups (**Table 3**). In addition, a negative effect of *K-ras* mutation on the OS of NSCLC patients with EGFR-TKI treatment was observed in all subgroups, which further confirmed the robustness of the general result.

**Table 3:** Results of subgroup analysis of pooled HRs for OS of patients harboring *K-ras* mutation with EGFR-TKI treatment

Subgroups	No. of study	Heterogeneity			Fixed model			Random model	
		*I*^2^ (%)	*P*		HR (95% CI)	*P*		HR (95% CI)	*P*
Concomitant with EGFR mutation									
No	5	19.5	0.290		1.79 (1.35-2.36)	0.000		1.83 (1.31-2.02)	0.000
Yes	2	73.4	0.052		1.37 (0.98-1.92)	0.069		1.53 (0.75-3.12)	0.246
NR	3	0.0	0.477		1.55 (1.09-2.21)	0.013		1.55 (1.09-2.21)	0.013
Previous treatment									
CT	7	12.9	0.331		1.73 (1.37-2.18)	0.000		1.73 (1.34-2.23)	0.000
Combination	2	0.0	0.406		1.84 (1.17-2.90)	0.009		1.84 (1.17-2.90)	0.009
NR	1	–	–		1.10 (0.80-1.80)	0.645		1.10 (0.80-1.80)	0.645
Mutation detection									
DS	3	14.2	0.312		1.58 (1.07-2.35)	0.022		1.59 (1.03-2.45)	0.037
DCE	2	0.0	0.643		1.63 (1.13-2.35)	0.008		1.63 (1.13-2.35)	0.008
RFLP	1	–	–		6.20 (1.58-24.31)	0.009		6.20 (1.58-24.31)	0.009
ME	1	–	–		2.29 (1.23-4.26)	0.009		2.29 (1.23-4.26)	0.009
Combination	2	56.5	0.129		1.38 (1.04-1.83)	0.025		1.37 (0.90-2.10)	0.146

CT, chemotherapy; DS, direct sequencing; DCE, denaturing capillary electrophoresis; ME, mutant-enrich sequencing; NR, not reported; RFLP, polymerase chain reaction-restriction fragment length polymorphism; HR, hazard ratio.

### Predictive value of *PIK3CA* mutation

**Figure 5:**
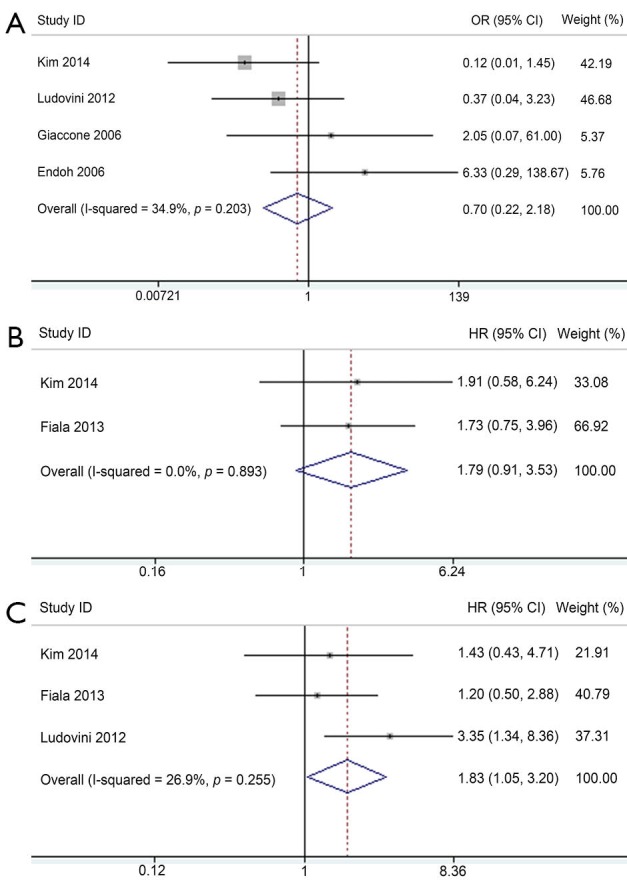
Meta-analysis of the predictive value of *PIK3CA* mutation. (A) Forest plots of OR and 95% CI for ORR; (B) Forest plots of HR and 95% CI for PFS; and (C) Forest plots of HR and 95% CI for OS. OR, odds ratio; HR, hazard ratio.

Five studies investigated the predictive role of *PIK3CA* mutation in NSCLC patients (**Table 2**). Among these, ORR data were available in four studies, PFS data in two studies, and OS data in three studies. *PIK3CA* mutant NSCLC patients exhibited similar response to EGFR-TKIs compared with wild-type patients with corresponding pooled OR of 0.70 (95% CI, 0.22-2.18) (**Figure 5A**). Fixed-effect model was used because heterogeneity across studies was not significant (*I*^2^=34.9%; *P*=0.203). The pooled HR of 1.79 (95% CI, 0.91-3.53) for PFS in a fixed-effect model (*I*^2^=0%; *P*=0.893) suggested that *PIK3CA* mutant NSCLC patients had similar PFS compared with wild-type patients when treated with EGFR-TKIs (**Figure 5B**). However, *PIK3CA* mutation showed a trend toward a significant adverse effect on OS with a pooled HR of 1.83 (95% CI, 1.05-3.20) in NSCLC patients treated with EGFR-TKIs (**Figure 5C**). Between-study heterogeneity was not significant; thus, the analysis was performed in the fixed-effect model (*I*^2^=26.9%; *P*=0.255).

Sensitivity analysis and publication bias of all above analyses was not performed because of the relatively limited eligible studies. Subgroup analysis was not conducted because of the relatively small size of included articles.

## Discussion

EGFR inhibitor elicits multiple downstream effects, primarily moderated by *RAS*/*RAF*/*MAPK* and *PI3K*/*AKT*/*mTOR* signaling pathways. Rational use of target therapy requires the optimal selection of patients whose tumors are dependent on the activation of these two pathways. The predictive value of gene mutations on these two pathways downstream of *EGFR* for EGFR-TKI treatment is gradually recognized. This meta-analysis reveals an independent predictive value of *K-ras* and *PIK3CA* genetic status on EGFR-TKI therapy.

Coincident with previous report, our results again demonstrated that NSCLC patients harboring *K-ras* mutation had poor response to EGFR-TKIs. Exclusion of possible duplicate study reduced publication bias significantly and did not alter the pooled result, thus proving the stability of our result. More importantly, we quantitatively demonstrated that such patients had shorter PFS and OS compared with wild-type patients. Given that heterogeneity was zero across the studies in the analysis of the impact of *K-ras* mutation on PFS of NSCLC patients treated with EGFR-TKIs, a trim-and-fill method was applied to adjust publication bias. The adjusted pooled HR did not alter significantly the primary result, suggesting the dependability of our results. Slight heterogeneity was observed in the meta-analysis of the impact of *K-ras* mutation on the OS of NSCLC patients treated with EGFR-TKIs. Subgroup analysis showed that if *K-ras* mutation is concomitant with EGFR mutation, previous treatment and mutation detection method (**Table 3**) might affect the result. However, a negative effect of *K-ras* mutation on the OS of NSCLC patients with the EGFR-TKI treatment was observed in all subgroups. All these results indicated the adverse impact of mutant *K-ras* on the response and survival outcomes of NSCLC patients treated with EGFR-TKIs. This adverse effect has been proved in other cancers^58^.

Mutant *PIK3CA* proteins increase catalytic activity resulting in enhanced downstream signaling and oncogenic transformation *in vitro*^59^. Preclinical data showed that introducing activated *PIK3CA* mutations into *EGFR*-mutated lung cancer cell lines confers resistance to EGFR-TKIs^60^. Consistent with this result, our analysis revealed significantly shorter OS, poor ORR, and shorter PFS in *PIK3CA* mutant NSCLC patients treated with EGFR-TKIs.

The most common mutation of *PIK3CA* was found in exons 9 and 20^61^, corresponding to the helical and kinase domains, respectively. The predictive value of *PIK3CA* as a negative biomarker for anti-EGFR response in colorectal cancer differed in exons 9 and 20^62^. However, similar study in NSCLC was rarely reported in published articles, and no clear evidence was obtained to show that the impact of mutations in exons 9 and 20 of *PIK3CA* on anti-EGFR response differ in NSCLC. Thus, further analysis of the predictive value of these two exons separately with enlarged samples size is needed to achieve definite conclusion.

Slight heterogeneity was observed in the analysis of the impact of *PIK3CA* mutation. Although subgroup analysis could not be conducted because of insufficient data, some diversity on whether *PIK3CA* mutation was concomitant with *EGFR* mutation, mutation detecting method, and data extraction method was observed. Coexistence of *PIK3CA* mutations with EGFR is frequent in lung cancer^15^^,^^63^^,^^64^. However, the predictive value of *PIK3CA* to anti-EGFR treatment in *EGFR* mutant or wild-type NSCLC is ambiguous at present. The accuracy and specificity of different mutation detection methods also varied, which led to different false positive and false negative rates^36^. Although extracting time-to-event data according to Tierney was preferable, it failed to circumvent the potential biases associated with relying on published data for meta-analysis as mentioned by the authors. Therefore, despite the slight heterogeneity of the included studies in the analysis of the impact of mutant *PIK3CA* on the response and survival outcomes of NSCLC patients, our result would be consolidated by increasing sample size.

Despite our efforts to provide an accurate and comprehensive analysis, limitations of our meta-analysis should be addressed. First, most of the included studies were retrospective. Second, not all published studies presented adjusted estimates or had been adjusted by similar potential confounders. Third, limited studies presented *PIK3CA* mutation data, in which only four studies provided ORR information, two studies provided PFS information, and three studies provided OS information. Thus, increasing sample size of studies will further increase the creditability of adverse effect of *PIK3CA* mutation on clinical prognosis of NSCLC patients receiving EGFR-TKI treatment.

In conclusion, this meta-analysis indicated that *K-ras* mutation is probably a valuable predictive biomarker for assessing the clinical response and survival outcomes of NSCLC patients treated with EGFR-TKIs. More importantly, similar trends for *PIK3CA* mutation were shown in this meta-analysis, although the trends in ORR and PFS were not significant. Increasing sample size of studies will further increase the creditability of adverse effect of *PIK3CA* mutation on the clinical prognosis of NSCLC patients receiving EGFR-TKI treatment. Mutations of *K-ras* and *EGFR* are usually mutually exclusive, and coexistence of mutation in *PIK3CA* and *EGFR* is common. Thus, determining the status of *K-ras* and *PIK3CA* is valuable to distinguish the optimal patients who will benefit from EGFR-TKI treatment.

## References

[1] Siegel RL Miller KD Jemal A. Cancer statistics, 2015. CA Cancer J Clin 2015;65:5-29. 25559415 10.3322/caac.21254

[2] Brambilla E Travis WD Colby TV Corrin B Shimosato Y. The new World Health Organization classification of lung tumours. Eur Respir J 2001;18:1059-1068. 11829087 10.1183/09031936.01.00275301

[3] Schiller JH Harrington D Belani CP Langer C Sandler A Krook J Comparison of four chemotherapy regimens for advanced non-small-cell lung cancer. N Engl J Med 2002;346:92-98. 11784875 10.1056/NEJMoa011954

[4] Pal SK Figlin RA Reckamp K. Targeted therapies for non-small cell lung cancer: an evolving landscape. Mol Cancer Ther 2010;9:1931-1944. 20571071 10.1158/1535-7163.MCT-10-0239 PMC3244351

[5] Wu YL Zhou C Hu CP Feng J Lu S Huang Y Afatinib versus cisplatin plus gemcitabine for first-line treatment of Asian patients with advanced non-small-cell lung cancer harbouring EGFR mutations (LUX-Lung 6): an open-label, randomised phase 3 trial. Lancet Oncol 2014;15:213-222. 24439929 10.1016/S1470-2045(13)70604-1

[6] Rosell R Carcereny E Gervais R Vergnenegre A Massuti B Felip E Erlotinib versus standard chemotherapy as first-line treatment for European patients with advanced EGFR mutation-positive non-small-cell lung cancer (EURTAC): a multicentre, open-label, randomised phase 3 trial. Lancet Oncol 2012;13:239-246. 22285168 10.1016/S1470-2045(11)70393-X

[7] Maemondo M Inoue A Kobayashi K Sugawara S Oizumi S Isobe H Gefitinib or chemotherapy for non-small-cell lung cancer with mutated EGFR. N Engl J Med 2010;362:2380-2388. 20573926 10.1056/NEJMoa0909530

[8] Ciardiello F Tortora G. EGFR antagonists in cancer treatment. N Engl J Med 2008;358:1160-1174. 18337605 10.1056/NEJMra0707704

[9] Thatcher JD. The Ras-MAPK signal transduction pathway. Sci Signal 2010;3:tr1. 20424265 10.1126/scisignal.3119tr1

[10] Gibbs JB Sigal IS Poe M Scolnick EM. Intrinsic GTPase activity distinguishes normal and oncogenic ras p21 molecules. Proc Natl Acad Sci U S A 1984;81:5704-5708. 6148751 10.1073/pnas.81.18.5704 PMC391779

[11] Samuels Y Wang Z Bardelli A Silliman N Ptak J Szabo S High frequency of mutations of the PIK3CA gene in human cancers. Science 2004;304:554. 15016963 10.1126/science.1096502

[12] Spaargaren M Bischoff JR McCormick F. Signal transduction by Ras-like GTPases: a potential target for anticancer drugs. Gene Expr 1995;4:345-356. 7549466 PMC6134362

[13] Schmelzle T Hall MN. TOR, a central controller of cell growth. Cell 2000;103:253-262. 11057898 10.1016/s0092-8674(00)00117-3

[14] Zoncu R Efeyan A Sabatini DM. mTOR: from growth signal integration to cancer, diabetes and ageing. Nat Rev Mol Cell Biol 2011;12:21-35. 21157483 10.1038/nrm3025 PMC3390257

[15] Li S Li L Zhu Y Huang C Qin Y Liu H Coexistence of EGFR with K-ras, or BRAF, or PIK3CA somatic mutations in lung cancer: a comprehensive mutation profiling from 5125 Chinese cohorts. Br J Cancer 2014;110:2812-2820. 24743704 10.1038/bjc.2014.210 PMC4037826

[16] Xu J He J Yang H Luo X Liang Z Chen J Somatic mutation analysis of EGFR, KRAS, BRAF and PIK3CA in 861 patients with non-small cell lung cancer. Cancer Biomark 2011-2012;10:63-69. 22430133 10.3233/CBM-2012-0233 PMC13016257

[17] Kim HR Cho BC Shim HS Lim SM Kim SK Chang J Prediction for response duration to epidermal growth factor receptor-tyrosine kinase inhibitors in EGFR mutated never smoker lung adenocarcinoma. Lung Cancer 2014;83:374-382. 24468202 10.1016/j.lungcan.2013.12.011

[18] Ludovini V Bianconi F Pistola L Pistola V Chiari R Colella R Optimization of patient selection for EGFR-TKIs in advanced non-small cell lung cancer by combined analysis of K-ras, PIK3CA, MET, and non-sensitizing EGFR mutations. Cancer Chemother Pharmacol 2012;69:1289-1299. 22302407 10.1007/s00280-012-1829-7

[19] Mao C Qiu LX Liao RY Du FB Ding H Yang WC K-ras mutations and resistance to EGFR-TKIs treatment in patients with non-small cell lung cancer: a meta-analysis of 22 studies. Lung Cancer 2010;69:272-278. 20022659 10.1016/j.lungcan.2009.11.020

[20] Burotto M Manasanch EE Wilkerson J Fojo T. Gefitinib and erlotinib in metastatic non-small cell lung cancer: a meta-analysis of toxicity and efficacy of randomized clinical trials. Oncologist 2015;20:400-410. 25795635 10.1634/theoncologist.2014-0154 PMC4391756

[21] Song Z Zhang Y. Efficacy of gefitinib or erlotinib in patients with squamous cell lung cancer. Arch Med Sci 2015;11:164-168. 25861304 10.5114/aoms.2013.39234 PMC4379363

[22] Tierney JF Stewart LA Ghersi D Burdett S Sydes MR. Practical methods for incorporating summary time-to-event data into meta-analysis. Trials 2007;8:16. 17555582 10.1186/1745-6215-8-16 PMC1920534

[23] Endoh H Yatabe Y Kosaka T Kuwano H Mitsudomi T. PTEN and PIK3CA expression is associated with prolonged survival after gefitinib treatment in EGFR-mutated lung cancer patients. J Thorac Oncol 2006;1:629-634. 17409929

[24] Giaccone G Gallegos Ruiz M Le Chevalier T Thatcher N Smit E Rodriguez JA Erlotinib for frontline treatment of advanced non-small cell lung cancer: a phase II study. Clin Cancer Res 2006;12:6049-6055. 17062680 10.1158/1078-0432.CCR-06-0260

[25] Han SW Kim TY Jeon YK Hwang PG Im SA Lee KH Optimization of patient selection for gefitinib in non-small cell lung cancer by combined analysis of epidermal growth factor receptor mutation, K-ras mutation, and Akt phosphorylation. Clin Cancer Res 2006;12:2538-2544. 16638863 10.1158/1078-0432.CCR-05-2845

[26] Cappuzzo F Ligorio C Janne PA Toschi L Rossi E Trisolini R Prospective study of gefitinib in epidermal growth factor receptor fluorescence in situ hybridization-positive/phospho-Akt-positive or never smoker patients with advanced non-small-cell lung cancer: the ONCOBELL trial. J Clin Oncol 2007;25:2248-2255. 17538169 10.1200/JCO.2006.09.4300

[27] Ichihara S Toyooka S Fujiwara Y Hotta K Shigematsu H Tokumo M The impact of epidermal growth factor receptor gene status on gefitinib-treated Japanese patients with non-small-cell lung cancer. Int J Cancer 2007;120:1239-1247. 17192902 10.1002/ijc.22513

[28] Jackman DM Yeap BY Lindeman NI Fidias P Rabin MS Temel J Phase II clinical trial of chemotherapy-naive patients > or = 70 years of age treated with erlotinib for advanced non-small-cell lung cancer. J Clin Oncol 2007;25:760-766. 17228019 10.1200/JCO.2006.07.5754

[29] van Zandwijk N Mathy A Boerrigter L Ruijter H Tielen I de Jong D EGFR and K-ras mutations as criteria for treatment with tyrosine kinase inhibitors: retro- and prospective observations in non-small-cell lung cancer. Ann Oncol 2007;18:99-103. 17060486 10.1093/annonc/mdl323

[30] Miller VA Riely GJ Zakowski MF Li AR Patel JD Heelan RT Molecular characteristics of bronchioloalveolar carcinoma and adenocarcinoma, bronchioloalveolar carcinoma subtype, predict response to erlotinib. J Clin Oncol 2008;26:1472-1478. 18349398 10.1200/JCO.2007.13.0062

[31] Schneider CP Heigener D Schott-von-Romer K Gutz S Laack E Digel W Epidermal growth factor receptor-related tumor markers and clinical outcomes with erlotinib in non-small cell lung cancer: an analysis of patients from german centers in the TRUST study. J Thorac Oncol 2008;3:1446-1453. 19057271 10.1097/JTO.0b013e31818ddcaa

[32] Wu CC Hsu HY Liu HP Chang JW Chen YT Hsieh WY Reversed mutation rates of K-ras and EGFR genes in adenocarcinoma of the lung in Taiwan and their implications. Cancer 2008;113:3199-3208. 18932251 10.1002/cncr.23925

[33] Zhu CQ da Cunha Santos G Ding K Sakurada A Cutz JC Liu N Role of K-ras and EGFR as biomarkers of response to erlotinib in National Cancer Institute of Canada Clinical Trials Group Study BR.21. J Clin Oncol 2008;26:4268-4275. 18626007 10.1200/JCO.2007.14.8924

[34] Zucali PA Ruiz MG Giovannetti E Destro A Varella-Garcia M Floor K Role of cMET expression in non-small-cell lung cancer patients treated with EGFR tyrosine kinase inhibitors. Ann Oncol 2008;19:1605-1612. 18467317 10.1093/annonc/mdn240 PMC7360138

[35] Boldrini L Ali G Gisfredi S Ursino S Baldini E Melfi F Epidermal growth factor receptor and K-ras mutations in 411 lung adenocarcinoma: a population-based prospective study. Oncol Rep 2009;22:683-691. 19724844 10.3892/or_00000488

[36] Marchetti A Milella M Felicioni L Cappuzzo F Irtelli L Del Grammastro M Clinical implications of K-ras mutations in lung cancer patients treated with tyrosine kinase inhibitors: an important role for mutations in minor clones. Neoplasia 2009;11:1084-1092. 19794967 10.1593/neo.09814 PMC2745674

[37] Varella-Garcia M Mitsudomi T Yatabe Y Kosaka T Nakajima E Xavier AC EGFR and HER2 genomic gain in recurrent non-small cell lung cancer after surgery: impact on outcome to treatment with gefitinib and association with EGFR and K-ras mutations in a Japanese cohort. J Thorac Oncol 2009;4:318-325. 19247083 10.1097/JTO.0b013e31819667a3 PMC3379811

[38] Amann JM Lee JW Roder H Brahmer J Gonzalez A Schiller JH Genetic and proteomic features associated with survival after treatment with erlotinib in first-line therapy of non-small cell lung cancer in Eastern Cooperative Oncology Group 3503. J Thorac Oncol 2010;5:169-178. 20035238 10.1097/JTO.0b013e3181c8cbd9 PMC3087978

[39] Douillard JY Shepherd FA Hirsh V Mok T Socinski MA Gervais R Molecular predictors of outcome with gefitinib and docetaxel in previously treated non-small-cell lung cancer: data from the randomized phase III INTEREST trial. J Clin Oncol 2010;28:744-752. 20038723 10.1200/JCO.2009.24.3030

[40] Tiseo M Rossi G Capelletti M Sartori G Spiritelli E Marchioni A Predictors of gefitinib outcomes in advanced non-small cell lung cancer (NSCLC): study of a comprehensive panel of molecular markers. Lung Cancer 2010;67:355-360. 19473722 10.1016/j.lungcan.2009.04.021

[41] Zhu YJ Xia Y Ren GJ Wang MZ Zeng X Zhang L. Efficacy and clinical/molecular predictors of erlotinib monotherapy for Chinese advanced non-small cell lung cancer. Chin Med J (Engl) 2010;123:3200-3205. 21163115

[42] Hirsch FR Kabbinavar F Eisen T Martins R Schnell FM Dziadziuszko R A randomized, phase II, biomarker-selected study comparing erlotinib to erlotinib intercalated with chemotherapy in first-line therapy for advanced non-small-cell lung cancer. J Clin Oncol 2011;29:3567-3573. 21825259 10.1200/JCO.2010.34.4929 PMC3179254

[43] Cadranel J Mauguen A Faller M Zalcman G Buisine MP Westeel V Impact of systematic EGFR and K-ras mutation evaluation on progression-free survival and overall survival in patients with advanced non-small-cell lung cancer treated by erlotinib in a French prospective cohort (ERMETIC project--part 2). J Thorac Oncol 2012;7:1490-1502. 22982650 10.1097/JTO.0b013e318265b2b5

[44] Metro G Chiari R Duranti S Siggillino A Fischer MJ Giannarelli D Impact of specific mutant K-ras on clinical outcome of EGFR-TKI-treated advanced non-small cell lung cancer patients with an EGFR wild type genotype. Lung Cancer 2012;78:81-86. 22770374 10.1016/j.lungcan.2012.06.005

[45] Murray S Karavasilis V Bobos M Razis E Papadopoulos S Christodoulou C Molecular predictors of response to tyrosine kinase inhibitors in patients with Non-Small-Cell Lung Cancer. J Exp Clin Cancer Res 2012;31:77. 22992338 10.1186/1756-9966-31-77 PMC3533816

[46] Campos-Parra AD Zuloaga C Manríquez ME Avilés A Borbolla-Escoboza J Cardona A KRAS mutation as the biomarker of response to chemotherapy and EGFR-TKIs in patients with advanced non-small cell lung cancer: clues for its potential use in second-line therapy decision making. Am J Clin Oncol 2015;38:33-40. 23538866 10.1097/COC.0b013e318287bb23

[47] Fiala O Pesek M Finek J Benesova L Bortlicek Z Minarik M. Gene mutations in squamous cell NSCLC: insignificance of EGFR, K-ras and PIK3CA mutations in prediction of EGFR-TKI treatment efficacy. Anticancer Res 2013;33:1705-1711. 23564819

[48] Kerner GS Schuuring E Sietsma J Hiltermann TJ Pieterman RM de Leede GP Common and rare EGFR and K-ras mutations in a Dutch non-small-cell lung cancer population and their clinical outcome. PLoS One 2013;8:e70346. 23922984 10.1371/journal.pone.0070346 PMC3726644

[49] Rotella V Fornaro L Vasile E Tibaldi C Boldrini L Chella A EGFR and K-Ras mutations in women with lung adenocarcinoma: implications for treatment strategy definition. J Exp Clin Cancer Res 2014;33:77. 25300933 10.1186/s13046-014-0077-6 PMC4198726

[50] Pao W Wang TY Riely GJ Miller VA Pan Q Ladanyi M K-ras mutations and primary resistance of lung adenocarcinomas to gefitinib or erlotinib. PLoS Med 2005; 2: e17. 15696205 10.1371/journal.pmed.0020017 PMC545207

[51] Hirsch FR Varella-Garcia M Bunn PA Jr Franklin WA Dziadziuszko R Thatcher N Molecular predictors of outcome with gefitinib in a phase III placebo-controlled study in advanced non-small-cell lung cancer. J Clin Oncol 2006;24:5034-5042. 17075123 10.1200/JCO.2006.06.3958

[52] Hirsch FR Varella-Garcia M Cappuzzo F McCoy J Bemis L Xavier AC Combination of EGFR gene copy number and protein expression predicts outcome for advanced non-small-cell lung cancer patients treated with gefitinib. Ann Oncol 2007;18:752-760. 17317677 10.1093/annonc/mdm003

[53] Loprevite M Tiseo M Chiaramondia M Capelletti M Bozzetti C Bortesi B Buccal mucosa cells as in vivo model to evaluate gefitinib activity in patients with advanced non small cell lung cancer. Clin Cancer Res 2007;13:6518-6526. 17975165 10.1158/1078-0432.CCR-07-0805

[54] Massarelli E Varella-Garcia M Tang X Xavier AC Ozburn NC Liu DD K-ras mutation is an important predictor of resistance to therapy with epidermal growth factor receptor tyrosine kinase inhibitors in non-small-cell lung cancer. Clin Cancer Res 2007;13:2890-2896. 17504988 10.1158/1078-0432.CCR-06-3043

[55] Felip E Rojo F Reck M Heller A Klughammer B Sala G A phase II pharmacodynamic study of erlotinib in patients with advanced non-small cell lung cancer previously treated with platinum-based chemotherapy. Clin Cancer Res 2008;14:3867-3874. 18559607 10.1158/1078-0432.CCR-07-5186

[56] Therasse P Arbuck SG Eisenhauer EA Wanders J Kaplan RS Rubinstein L New guidelines to evaluate the response to treatment in solid tumors. European Organization for Research and Treatment of Cancer, National Cancer Institute of the United States, National Cancer Institute of Canada. J Natl Cancer Inst 2000;92:205-216. 10655437 10.1093/jnci/92.3.205

[57] Miller AB Hoogstraten B Staquet M Winkler A. Reporting results of cancer treatment. Cancer 1981;47:207-214. 7459811 10.1002/1097-0142(19810101)47:1<207::aid-cncr2820470134>3.0.co;2-6

[58] Loupakis F Pollina L Stasi I Ruzzo A Scartozzi M Santini D PTEN expression and K-ras mutations on primary tumors and metastases in the prediction of benefit from cetuximab plus irinotecan for patients with metastatic colorectal cancer. J Clin Oncol 2009;27:2622-2629. 19398573 10.1200/JCO.2008.20.2796

[59] Kang S Bader AG Vogt PK. Phosphatidylinositol 3-kinase mutations identified in human cancer are oncogenic. Proc Natl Acad Sci U S A 2005;102:802-807. 15647370 10.1073/pnas.0408864102 PMC545580

[60] Engelman JA Mukohara T Zejnullahu K Lifshits E Borras AM Gale CM Allelic dilution obscures detection of a biologically significant resistance mutation in EGFR-amplified lung cancer. J Clin Invest 2006;116:2695-2706. 16906227 10.1172/JCI28656 PMC1570180

[61] Abubaker J Bavi P Al-Harbi S Ibrahim M Siraj AK Al-Sanea N Clinicopathological analysis of colorectal cancers with PIK3CA mutations in Middle Eastern population. Oncogene 2008;27:3539-3545. 18193083 10.1038/sj.onc.1211013

[62] De Roock W Claes B Bernasconi D De Schutter J Biesmans B Fountzilas G Effects of K-ras, BRAF, NRAS, and PIK3CA mutations on the efficacy of cetuximab plus chemotherapy in chemotherapy-refractory metastatic colorectal cancer: a retrospective consortium analysis. Lancet Oncol 2010;11:753-762. 20619739 10.1016/S1470-2045(10)70130-3

[63] Arcila ME Nafa K Chaft JE Rekhtman N Lau C Reva BA EGFR exon 20 insertion mutations in lung adenocarcinomas: prevalence, molecular heterogeneity, and clinicopathologic characteristics. Mol Cancer Ther 2013;12:220-229. 23371856 10.1158/1535-7163.MCT-12-0620 PMC3714231

[64] Chaft JE Arcila ME Paik PK Lau C Riely GJ Pietanza MC Coexistence of PIK3CA and other oncogene mutations in lung adenocarcinoma-rationale for comprehensive mutation profiling. Mol Cancer Ther 2012;11:485-491. 22135231 10.1158/1535-7163.MCT-11-0692 PMC3593239

